# Categorisation of continuous covariates for stratified randomisation: How should we adjust?

**DOI:** 10.1002/sim.10060

**Published:** 2024-03-15

**Authors:** Thomas R. Sullivan, Tim P. Morris, Brennan C. Kahan, Alana R. Cuthbert, Lisa N. Yelland

**Affiliations:** 1 Women and Kids Theme, South Australian Health and Medical Research Institute, Adelaide, South Australia, Australia; 2 School of Public Health, The University of Adelaide, Adelaide, South Australia, Australia; 3 MRC Clinical Trials Unit, UCL, London, UK

**Keywords:** categorise, covariate adjustment, dichotomise, randomised trial, stratification variable

## Abstract

To obtain valid inference following stratified randomisation, treatment effects should be estimated with adjustment for stratification variables. Stratification sometimes requires categorisation of a continuous prognostic variable (eg, age), which raises the question: should adjustment be based on randomisation categories or underlying continuous values? In practice, adjustment for randomisation categories is more common. We reviewed trials published in general medical journals and found none of the 32 trials that stratified randomisation based on a continuous variable adjusted for continuous values in the primary analysis. Using data simulation, this article evaluates the performance of different adjustment strategies for continuous and binary outcomes where the covariate-outcome relationship (via the link function) was either linear or non-linear. Given the utility of covariate adjustment for addressing missing data, we also considered settings with complete or missing outcome data. Analysis methods included linear or logistic regression with no adjustment for the stratification variable, adjustment for randomisation categories, or adjustment for continuous values assuming a linear covariate-outcome relationship or allowing for non-linearity using fractional polynomials or restricted cubic splines. Unadjusted analysis performed poorly throughout. Adjustment approaches that misspecified the underlying covariate-outcome relationship were less powerful and, alarmingly, biased in settings where the stratification variable predicted missing outcome data. Adjustment for randomisation categories tends to involve the highest degree of misspecification, and so should be avoided in practice. To guard against misspecification, we recommend use of flexible approaches such as fractional polynomials and restricted cubic splines when adjusting for continuous stratification variables in randomised trials.

## 1 Introduction

Stratified randomisation is widely used in clinical trials to guard against chance imbalances between treatment groups in key prognostic variables.^1–4^ To implement stratified randomisation, participants are grouped into strata defined by one or more baseline prognostic variables, termed *stratification variables*, and assigned to treatment groups according to a stratum-specific randomisation schedule, typically using permuted blocks. By ensuring balance on stratification variables, stratified randomisation can enhance trial credibility and allow for treatment effects to be estimated with greater precision than under simple randomisation, particularly in smaller trials.^5–8^ To realise the precision gains of stratified randomisation and obtain valid inference, treatment effects should be estimated with adjustment for variables used to stratify the randomisation.^5,6,9–12^ Since stratified randomisation induces a correlation between treatment groups, failure to adjust for stratification variables when estimating treatment effects can lead to overly wide confidence intervals, type I error rates less than *α*, and reduced statistical power.^7,9,12^

Prognostic variables in clinical trials are often continuous, for example age or the baseline value of a continuous outcome. Such variables can only be used in a stratified randomisation scheme if first categorised (eg, age < 40 years or ≥40 years). When a continuous prognostic variable is categorised for stratified randomisation, the need to adjust for stratification variables in the analysis raises the question of whether the adjustment should be based on the categories used for randomisation or the underlying continuous values. Adjusting for randomisation categories of a continuous stratification variable may be appealing as it matches the randomisation design and ensures that any correlation induced by stratification is adequately addressed. Conversely, adjusting for the underlying continuous values avoids the loss of information associated with categorising and may offer power advantages by potentially better approximating the data generation process, however choices must be made regarding the assumed covariate-outcome relationship.

The topic of covariate adjustment has been explored at length in the context of trials using simple randomisation. For continuous outcomes, a widely discussed approach is the ANCOVA-type estimator, which involves fitting a linear model for the outcome given treatment and one or more baseline covariates. In trials involving 1:1 simple randomisation and a continuous baseline covariate, the ANCOVA estimator is known to be (1) the most powerful estimator of the treatment effect when the linear model is correctly specified and (2) consistent otherwise under arbitrary misspecification, including misspecification of the covariate-outcome relationship.^13,14^ Consequently, some authors suggest keeping covariate values as continuous in the analysis to maximise power,^15^ while others advocate using simple functional forms for modelling the covariate-outcome relationship, including the use of categories, given the robustness properties of ANCOVA.^11^ The situation is more complex for non-collapsible measures such as the odds ratio, but adjustment for continuous covariate values may again be preferred for the potential improvements in power.^15^ Whether these recommendations should apply to continuous covariates categorised for stratification is less clear. Although recent work has demonstrated links between the statistical properties of various estimators under both simple and stratified randomisation,^7,8^ practical guidance on how to handle continuous covariates used for stratified randomisation in the analysis remains lacking.

As well as increasing the precision of treatment effect estimates, covariate adjustment can play an important role in addressing missing outcome data, a problem that affects most randomised trials. For example, when missing data are confined to a univariate outcome and the probability of missing data depends only on randomised group and covariate values (ie, outcome data missing at random), the ANCOVA-type estimator produces unbiased and efficient estimates of the treatment effect when the linear model is correctly specified, whereas the unadjusted estimator is biased in general.^16–18^ Unfortunately, the ability of ANCOVA to address missing outcome data under model misspecification is less well understood, and key guidance documents on covariate adjustment, including those of the Food & Drug Administration^19^ and the European Medicines Agency,^11^ do not offer advice for settings with missing outcome data. The issue of model misspecification with missing outcome data is particularly salient to trials involving continuous stratification variables, as these variables may be chosen not just for being highly prognostic of the outcome but also for their ability to predict missing data. Additionally, trial statisticians may be more accepting of a misspecified analysis model in this context (eg, involving adjustment for randomisation categories of a continuous stratification variable) given the general notion that the analysis should match the randomisation design.

In this article we compare different covariate adjustment approaches for the estimation of treatment effects in trials where a continuous covariate has been categorised for use as a stratification variable. We explore settings involving both continuous and binary outcomes, where the covariate-outcome relationship (via the link function) is either linear or non-linear, and where data on the outcome are complete or incomplete. We predominantly focus on settings where the treatment effect is constant across values of the stratification variable, although consideration is also given to estimating average treatment effects in the presence of treatment effect modification. The remainder of the article is structured as follows. Section 2 presents a literature review to assess how often continuous variables are used for stratification and how such variables are handled in the analysis. In Section 3 we introduce notation and outline simulation methods for evaluating the performance of different adjustment approaches, the results of which are presented in Section 4. In Section 5 we apply the competing adjustment approaches to data from a real trial, where randomisation was stratified by a continuous prognostic variable. Finally, conclusions and general recommendations are provided in Section 6.

## 2 Review of Current Practice

To assess how frequently continuous variables are used for stratified randomisation and approaches to the primary analysis in these trials, we (authors TRS and ARC) reviewed original reports of randomised confirmatory trials published over a four-month period between April 1st 2022 and July 31st 2022 in four leading general medical journals (Lancet, The BMJ, Journal of the American Medical Association and New England Journal of Medicine). For eligible articles, we extracted information on the method of randomisation, variables used for stratified randomisation, and the method of covariate adjustment in the primary analysis. Details reported in supplementary materials and web appendices were also evaluated in the review.

In total, 105 eligible trials were identified (see Supplementary materials for included articles), of which 79 (75%) used stratified randomisation and 32 (30%) involved stratification by one or more underlying continuous variables (total 35 continuous variables). Most continuous variables were categorised into two levels for the purpose of stratified randomisation (31 out of 35 variables), with age the most common continuous stratification variable (17 trials). Among the 32 trials involving one or more continuous stratification variables, 31 based primary conclusions on the results of an overall treatment group comparison. In the remaining trial, the primary analysis involved subgroup comparisons in randomisation categories of the continuous stratification variable, albeit an overall treatment group comparison was still provided. For the 32 overall treatment group comparisons, an unadjusted analysis was performed in 10 (31%), which may have resulted in overly conservative confidence intervals and significance tests for the treatment effect; 8 trials used a method which did not allow for straightforward adjustment (eg, a chi-square or an exact test), while the remaining 2 did not adjust despite using a method that could accommodate adjustment. Other approaches included adjusting for the randomisation categories in a regression model (12 trials, 38%) or performing a stratified analysis according to the randomisation categories (eg, Cochran-Mantel-Haenszel test or stratified log rank test, 7 trials, 22%). In three trials the adjustment approach was unclear (uncertain whether adjustment performed, which covariates were adjusted for, or whether the stratification variable was adjusted for using randomisation categories or underlying continuous values). Importantly, none of the 32 articles reported adjusting for the underlying continuous values of the stratification variable in the primary analysis.

## 3 Simulation Study Methods

A simulation study following the ADEMP (aims, data generating mechanisms, estimands, methods, performance measures) framework^20^ was undertaken to investigate the performance of alternative adjustment approaches in trials involving stratified randomisation by an underlying continuous variable. Our aim was to assess the performance of direct adjustment approaches for estimating treatment effects, where the continuous stratification variable is included in some form as a covariate for adjustment in a regression model for the outcome. We focus on the common setting of 1:1 randomisation, where the ordinary least squares estimator from a linear regression model with adjustment for stratification categories (without interaction terms) is known to produce consistent point and variance estimates for the treatment effect under arbitrary misspecification.^7,8,21^ As the precision of treatment effect estimates was anticipated to depend on the degree of misspecification of the relationship between the stratification variable and the outcome, we considered settings involving both linear and non-linear relationships. We also explored settings involving both continuous and binary outcomes analysed using linear and logistic models respectively, and where data on the outcome were complete or incomplete. All calculations were performed using Stata version 18 (StataCorp, College Station, Texas, USA), with the code required to reproduce the results of the simulation study provided in the Supplementary materials. For each simulation scenario, 5000 datasets were generated.

### 3.1 Continuous outcome, complete data

Data generating mechanisms in the simulation study were largely based on earlier work by Kahan and colleagues,^15^ who explored adjustment for continuous covariates in trials with simple randomisation. For the *i*th participant in each simulated dataset (*i* = 1–200), a continuous baseline variable *X*_*i*_~*N*(0, 1) was generated and then categorised into $X_{i}^{strat}$ for the purpose of stratified randomisation, with $X_{i}^{strat}=0$ if *X*_*i*_ < 0 and $X_{i}^{strat}=1$ if *X*_*i*_ ≥ 0. The sample size of 200 corresponds to the median sample size observed for continuous outcomes in a previous review of trials.^1^ Next, participants were randomised to treatment group *T*_*i*_ (0 = control and 1 = intervention), stratifying on *X*^*strat*^ and using randomly permuted blocks of size 4 within strata. Outcome data were then generated from the model *Y*_*i*_ = *β*_0_ + *β*_1_*T*_*i*_ + *β*_2_*f* (*X*_*i*_) + *e*_*i*_, with *f* (.) a transformation and *e*_*i*_~*N*(0, 1) a random error term (independent and identically distributed). The intercept term β_0_ is not important for assessing performance in estimating treatment effects and so was arbitrarily set to 0 in all simulations. The treatment effect *β*_1_ was set to 0 (to assess type-I error) or 0.4 based on power considerations (100 observations per group provides approximately 80% power to detect a standardised mean difference of 0.4 between treatment groups using a *t*-test, α= 0.05 two-tailed).

**Figure 1:**
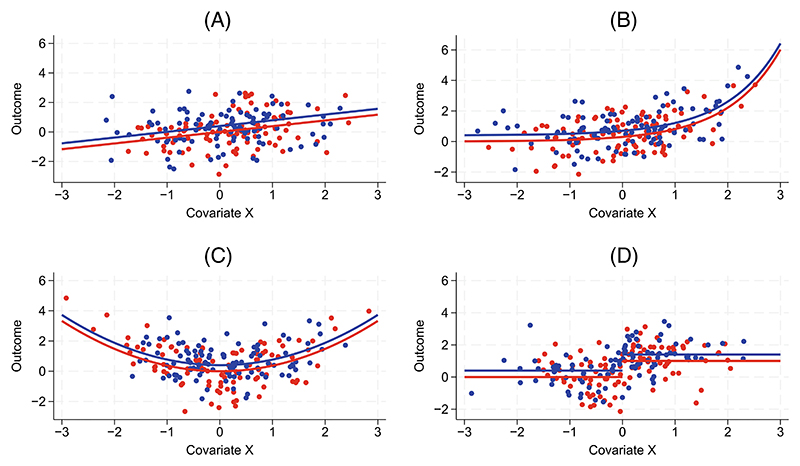
Relationships between the continuous covariate and outcome in simulation scenarios involving moderate covariate-outcome relationships for (A) *f* (*X*) = *X*, (B) *f* (*X*) = *e*^*X*^, (C) *f* (*X*) = *X*^2^ and (D) *f* (*X*) = *X*^*strat*^. Dots depict simulated values from a single dataset of 200 observations and lines the true relationships used for data generation. Blue indicates the intervention group and red the control group.

For the transformation *f* (.), the following scenarios were considered to provide a broad range of shapes for the covariate-outcome relationship: (a) linear relationship with the outcome, *f* (*X*) = *X*; (b) exponential relationship, *f* (*X*) = *e^X^* ; (c) quadratic relationship, *f* (*X*) = *X*^2^; and (d) step function relationship according to the stratification categories, *f* (*X*) = *X^strat^*. Performance under linear, exponential, and quadratic relationships was of most interest, as these functions have been reported in many clinical contexts.^22,23^ While step function relationships are unlikely to be encountered in practice due to their unrealistic shape, they were included to explore the performance of analysis methods that treat the mean function as ‘smooth’ under misspecification. The covariate effect *β*_2_ was chosen so that in each scenario a change from the 10th to the 90th percentile in *f* (*X*) increased the outcome by one or two units (equivalently standard deviations in *e*_*i*_), denoted as *moderate* and *strong* covariate-outcome relationships, respectively. This led to *β*_2_ values of 0.39 or 0.78 for *f* (*X*) = *X*, 0.30 or 0.60 for *f* (*X*) = *e*^*X*^, 0.37 or 0.74 for *f* (*X*) = *X*^2^, and 1 or 2 for *f* (*X*) = *X*^*strat*^. Moderate covariate-outcome relationships are depicted graphically in Figure 1. Note that for *f* (*X*) = *X*, the coefficient values of 0.39 and 0.78 produced correlations with the outcome (ie, corr(*X, Y*|*T*)) of 0.36 and 0.61, respectively.

The estimand of interest was the expected difference in potential outcomes between the intervention and control treatments with true value *β*_1_. To estimate this estimand, generated datasets were analysed using linear regression, with the following approaches to adjust for the stratification variable: (1) no adjustment; (2) adjustment for the randomisation categories *X*^*strat*^; (3) adjustment for continuous *X* assuming a linear relationship with the outcome; (4) adjustment for continuous *X* using two-term fractional polynomials; and (5) adjustment for continuous *X* using restricted cubic splines with 5 knots placed using the standard percentile method. Two-term fractional polynomials (denoted FP-2) and restricted cubic splines were chosen for investigation as these approaches have previously been shown to work well in trials involving simple randomisation and are easily pre-specified.^15^ For the restricted cubic splines, 5 knots were chosen as this number has been recommended previously.^15,24^ Model-based SEs were used and confidence intervals were constructed using the *t*-distribution. The performance measures of interest included the percentage bias in estimated treatment effects, coverage of 95% confidence intervals (or equivalently type-I error rate for *β*_1_ = 0), empirical SE and power (for *β*_1_ = 0.4). Monte Carlo SEs for these performance measures were also calculated.^25^

### 3.2 Continuous outcome, missing data

The simulation study described in Section 3.1 was repeated with incomplete outcome data. We considered settings where the probability of missing outcome data depended on both the stratification variable and treatment group (ie, outcome data missing at random), such that correct specification of a covariate-adjusted complete case analysis model would be expected to lead to valid inference. Specifically, outcome values were randomly set to missing according to the model logit *P* (*Y*_*i*_ missing) = *γ* + log(1.5)*T*_*i*_ + log(1.5)*X*_*i*_ + log(1.5)*X*_*i*_*T*_*i*_. Under this model the odds of missing data were 1.5 times higher per SD increase in *X* in the control group, and 2.25 times higher per SD increase in *X* in the treatment group. The intercept term *γ* was set to produce 30% missing outcome data overall and analyses were based on the complete data (complete case analysis). A relatively high (but realistic) missing data rate of 30% was chosen so that any performance deficits due to missing data were easily identified. To further explore results in the missing outcome data setting, sensitivity analyses were performed under simple randomisation instead of stratified randomisation, and with multiple imputation used to address missing data instead of complete case analysis (*m* = 30 imputations with the imputation model matching the analysis model). Performance under a missing completely at random mechanism was not considered since missing data can be safely ignored without introducing bias under this more restrictive mechanism (and conclusions would mirror those from complete data settings).

### 3.3 Continuous outcome with a treatment by covariate interaction, complete and missing data

The implications of ignoring an interaction effect between the continuous stratification variable and treatment group on treatment effect estimation were investigated by modifying the data generating mechanism in Section 3.1 so that the covariate-outcome relationship applied only in the intervention group, that is, *Y*_*i*_ = *β*_1_*T*_*i*_ + *β*_3_*f* (*X*_*i*_)*T*_*i*_ + *e*_*i*_. As interaction effects were not a focus of the paper, for brevity we restricted attention to the subset of settings with a treatment effect *β*_1_ of 0.4 and a linear covariate outcome-relationship, that is, *f* (*X*) = *X* with *β*_3_ = 0.39 or 0.78 for moderate and strong relationships, respectively. We considered settings with both complete and missing outcome data, with 30% missing data induced using the mechanism in Section 3.2. Noting recommendations to omit interaction terms from primary analysis models,^10,11^ the average treatment effect (with true value of 0.4 in this setup) was estimated using the five adjustment approaches described in Section 3.1, fitted without inclusion of an interaction term between *X* and *T*.

### 3.4 Binary outcome, complete and missing data

To investigate whether the performance of the adjustment approaches depends on the type of the outcome, we extended the simulation study to consider binary *Y*_*i*_ generated from a logistic model and assuming interest in a conditional odds ratio or a marginal risk difference for the effect of treatment, with the latter estimated via standardisation. Methods for evaluation included logistic regression with the same five adjustment approaches that were used for the continuous outcome setting. We also explored the performance of the Cochran-Mantel-Haenszel test with stratification by *X*^*strat*^, given the widespread use of this approach in the literature review. As before, we considered settings with both complete and incomplete outcome data. Due to similarities with the simulation study for continuous outcome data, further details of the binary simulation study are provided in the Supplementary materials.

## 4 Simulation Study Results

### 4.1 Continuous outcome, complete data

When the treatment effect *β*_1_ was set to 0, all methods that adjusted for the stratification variable in some form were unbiased (results not shown) and exhibited appropriate type-I error rates (Table 1). While the unadjusted analysis was also unbiased, this approach produced type-I error rates below the nominal 0.05 level for linear, exponential, and the unrealistic step function relationships, as expected based on previous research demonstrating the need to adjust for stratification variables to avoid overly conservative inference. Problems with type I error were not evident for the unadjusted analysis under the quadratic covariate-outcome relationship (ie, *f* (*X*) = *X*^2^), where the expected mean of the outcome was the same across strata (and hence where the relationship between treatment group means induced by stratification did not lead to correlated means).

**Figure 2:**
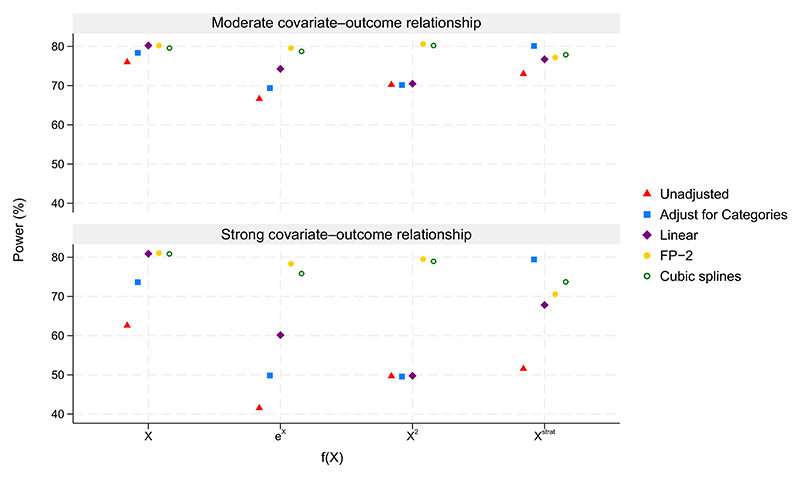
Power in scenarios with a continuous outcome, complete data, and treatment effect of 0.4. The maximum Monte Carlo SE across all methods and scenarios was 0.71%.

When the treatment effect β_1_ was set to 0.4, all analysis methods again produced unbiased treatment effect estimates throughout, with only the unadjusted analysis exhibiting any problems with coverage (coverage 0.977 for *f* (*X*) = *X*, 0.973 for *f* (*X*) = *e*^*X*^ and 0.995 for *f* (*X*) = *X*^*strat*^ under a strong covariate-outcome relationship; results not shown). The unadjusted analysis was also the least powerful approach in all scenarios (see Figure 2). Adjusting for stratification categories was associated with higher empirical SEs (Table 2) and lower power (Figure 2) than competing adjustment approaches under linear, exponential, and quadratic covariate-outcome relationships, but was optimal for these performance measures when data were generated using the unrealistic step function relationship. Assuming a linear relationship in the analysis was optimal when *f* (*X*) = *X* and more powerful than adjusting for stratification categories when *f* (*X*) = *e*^*X*^, but did not lead to any improvements over adjusting for categories under *f* (*X*) = *X*^2^ (since the quadratic function is symmetrical around the threshold for forming stratification categories the models are equally misspecified). Both the FP-2 and cubic spline approaches performed well in settings involving a linear, exponential, and quadratic covariate-outcome relationship, producing similar results to adjustment assuming a linear relationship when *f* (*X*) = *X* and noticeably out-performing this approach for *f* (*X*) = *e*^*X*^ and *f* (*X*) = *X*^2^. Only the unrealistic step function showed any performance deficits for the FP-2 and cubic spline approaches. Overall, the loss of precision and power associated with misspecification of the covariate-outcome relationship was more pronounced for strong than moderate covariate-outcome relationships.

### 4.2 Continuous outcome, missing data

**Figure 3:**
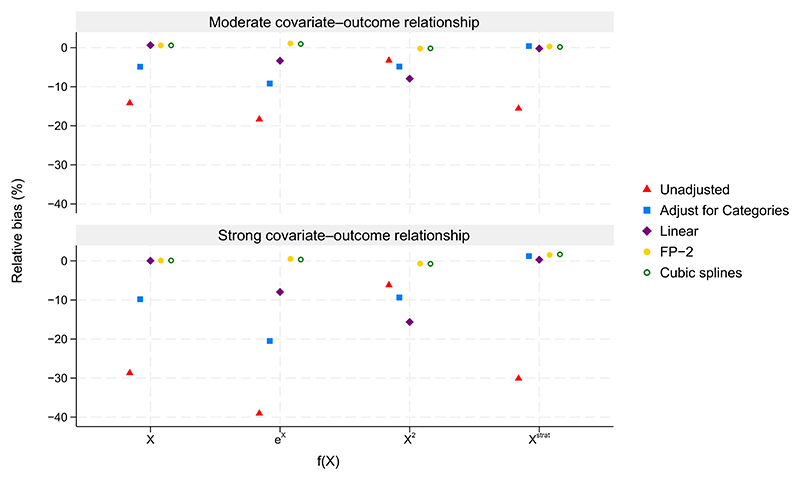
Percentage bias in scenarios with a continuous outcome, 30% missing outcome data, and treatment effect of 0.4. The maximum Monte Carlo SE across all methods and scenarios was 0.86%.

Figure 3 displays percentage bias in the treatment effect estimate when the true effect was 0.4 and there were 30% missing outcome data. Unsurprisingly the unadjusted analysis produced biased treatment effect estimates throughout, a consequence of data missing not at random when failing to condition on the covariate *X*, a cause of the data being missing. Interestingly, misspecification of the covariate-outcome relationship in an adjusted analysis was also associated with biased treatment effect estimates. Adjustment for stratification categories led to biased estimates under linear, exponential, and quadratic covariate-outcome relationships, as did assuming a linear relationship with *X* when the true covariate-outcome relationship was exponential or quadratic. Conversely, both the FP-2 and cubic spline approaches produced unbiased treatment effect estimates with nominal coverage (coverage results not shown) for linear, exponential, and quadratic covariate-outcome relationships. All methods of adjustment produced unbiased treatment effect estimates under the unrealistic step function relationship, despite model misspecification when assuming a linear covariate-outcome relationship or using FP-2 or cubic spline approaches. Overall, the magnitude of the bias due to mis-specification was more pronounced for strong than moderate covariate-outcome relationships. It should be noted that bias due to model misspecification when conditioning on fully observed causes of missing outcome data is not a special feature of stratified randomisation. In sensitivity analyses substituting simple randomisation in place of stratified randomisation, similar results were observed (see Supplementary materials Figure S1). Performance was also similar when multiple imputation was used to address missing data instead of complete case analysis, as expected for missingness confined to a univariate outcome (results not shown).

### 4.3 Continuous outcome with a treatment by covariate interaction, complete and missing data

In complete data settings involving an interaction effect between the continuous stratification variable and treatment group, all methods produced unbiased treatment effect estimates with confidence intervals exhibiting nominal coverage.

While the unadjusted analysis remained the least powerful approach throughout, there was little difference in power across methods of adjustment, a product of each analysis model being misspecified to some degree through omission of the interaction term (Table 3). In contrast, such misspecification led to biased treatment effect estimates in settings with missing outcome data, most prominent for unadjusted analysis but also evident with all methods of adjustment. The magnitude of the bias due to misspecification was more pronounced for the strong than the moderate covariate-outcome relationship (Table 3).

### 4.4 Binary outcome, complete and missing data

Full results of the binary simulation study are provided in the Supplementary materials. Overall, the pattern of results for the risk difference followed those of the mean difference in the continuous outcome data setting, while some changes were observed for the odds ratio due to non-collapsibility. Briefly, all analysis methods were unbiased under a null treatment effect, regardless of whether interest concerned the conditional odds ratio or the risk difference, with only the unadjusted analysis producing overly conservative type-I error rates. When the treatment effect was non-null (conditional odds ratio of 1.5), misspecification of the covariate-outcome relationship was associated with losses in power and attenuated estimates of the conditional odds ratio. This is an expected feature of imperfect conditioning with non-collapsible measures, where the true value is some average of the fully-conditional and fully-marginal measures. Such attenuation was no longer evident, however, when standardisation was used following logistic regression to estimate the risk difference (all methods unbiased), as expected given the risk difference is a marginal measure. When missing data were induced in the outcome, misspecification of the covariate-outcome relationship led to biased estimates of both the conditional odds ratio and the risk difference. Overall, the FP-2 and cubic spline approaches performed best (highest power and unbiased with missing outcome data) for the exponential and quadratic covariate-outcome relationships and performed similarly to adjustment assuming a linear relationship for *f* (*X*) = *X*. Adjusting for stratification categories (in logistic regression or using the Cochran-Mantel-Haenszel test) performed poorly, except where the covariate-outcome relationship followed an unrealistic step function.

## 5 Example Analysis of the Dino Trial

To illustrate the impact of different covariate adjustment approaches in practice, we consider data from the Docosahexaenoic Acid for the Improvement of Neurodevelopmental Outcome in Preterm Infants (DINO) trial. This trial was conducted in five Australian hospitals between 2001 and 2007.^26^ 657 preterm infants born less than 33 weeks’ gestation were randomised within 5 days of commencing enteral feeds to receive a high docosahexaenoic acid (DHA; approximately 1% of total fatty acids) diet or a standard DHA (approximately 0.3% of total fatty acids) diet from randomisation to 40 weeks’ post-menstrual age. Randomisation was stratified by hospital, sex and birth weight using randomly permuted blocks of size 4, with birth weight categorised as <1250 g or ≥1250 g (approximately 45% and 55% of infants fell in each category, respectively).

Here we consider analysis of the binary secondary outcome of need for supplemental oxygen support for chronic lung disease by 36 weeks’ gestation, the results of which motivated subsequent trials on the effects of DHA supplementation on lung disease.^27,28^ As infants from a multiple birth were randomised according to the sex and birth weight of the first-born infant, for illustration purposes we exclude 115 second and subsequent born infants from a multiple birth. Excluding a further 4 infants with missing data from the analysis, 47/269 (17.5%) required supplemental oxygen in the high DHA group compared to 65/269 (24.2%) in the standard DHA group. As shown in Table 4, failing to adjust for stratification variables in a logistic regression model for supplemental oxygen was associated with the most conservative inference for the treatment effect (whether expressed as an odds ratio or risk difference). Compared to adjusting for birth weight randomisation categories or stratifying on these categories in a Cochran-Mantel-Haenszel test, treating birth weight as continuous in the analysis increased the magnitude of the conditional odds ratio estimate and slightly improved the precision of the risk difference estimate. No further change was seen with the FP-2 and cubic spline approaches, as expected given the relationship between birth weight and the log odds of supplemental oxygen appeared to be approximately linear (see Supplementary materials, Figure S5).

Overall, the example analysis highlights the value of adjusting for underlying continuous values of a stratification variable and shows that the FP-2 and cubic spline approaches perform similarly to adjustment assuming a linear relationship for linear covariate-outcome relationships. This agrees with simulation results, which demonstrated that the FP-2 and cubic spline approaches offer the most benefit in settings involving non-linear covariate-outcome relationships.

## 6 Discussion

In this article we explored the use of adjustment approaches for handling continuous covariates used for stratification in the analysis of randomised trials. Our review of recent trials published in leading general medical journals found that stratification based on an underlying continuous variable is common and analyses are typically performed based on the randomisation categories or using an unadjusted model, yet these approaches did not perform well in our simulation study. Consistent with previous findings for simple randomisation,^15^ we showed that misspecification of the covariate-outcome relationship can lead to substantial reductions in power following stratified randomisation, based on decreased precision for collapsible effect measures (mean difference or risk difference) and attenuation of the conditional effect estimate for non-collapsible effect measures (odds ratio). Concerningly, we demonstrated that misspecification of the covariate-outcome relationship can introduce bias in settings where the stratification variable causes missingness in outcome data. We therefore advocate adjustment for the underlying continuous values of stratification variables rather than adjustment for the categories used for stratification. Unless there is a strong a priori reason for assuming a linear covariate-outcome relationship (via the link function), to minimise the possibility of misspecification we recommend use of flexible approaches such as two-term fractional polynomials and restricted cubic splines when adjusting for continuous stratification variables in randomised trials.

Guidance from the European Medicines Agency on covariate adjustment in randomised trials recommends adopting a simple functional form for a continuous covariate when its relationship with the outcome is not well understood, either by assuming linearity or forming a small number of categories.^11^ For trials stratified by a continuous covariate, this advice could imply adjustment for the randomisation categories, since categories would be defined at the time of writing the statistical analysis plan and prior thought would have been given to appropriate thresholds for forming categories. Excepting rare cases where randomisation categories might work well to capture the relationship between a continuous covariate and the outcome (ie, where the underlying relationship follows a step function and the thresholds for forming categories match those of the step function), we again caution against such adjustment due to the potential for lost power and introduction of bias in settings with missing outcome data.

Under 1:1 stratified randomisation and assuming complete data, the ordinary least squares estimator from a linear regression model with adjustment for stratification categories (excluding interaction terms) is known to produce consistent point and variance estimates for the treatment effect under arbitrary misspecification.^7,8,21^ We observed such performance for continuous outcomes in the simulation study under misspecification of the covariate-outcome relationship, including omission of a treatment by covariate interaction term, regardless of the specific method of adjustment employed. Results differed in settings with missing outcome data, however. Consistent with previous findings,^16–18^ we observed unbiased treatment effect estimates when baseline causes of missing outcome data were adjusted for in a correctly specified complete case analysis model. However, when the analysis model was misspecified, covariate adjustment was no longer entirely effective in avoiding bias due to missing outcome data. In misspecifying the covariate-outcome relationship, in effect we do not fully condition on the covariate, which in the simulation study equated to violating an assumption that outcome data were missing at random conditional on covariates. The findings are consistent with recent work highlighting the potential for analysis model misspecification to produce bias with complete case analysis or multiple imputation when data are missing at random and missingness does not depend on the outcome; settings where these methods would otherwise be expected to lead to valid inference.^29^ The potential for bias in randomised trials with outcome data missing at random due solely to model misspecification has not been highlighted previously. Although especially relevant to continuous stratification variables, the potential for biased estimation with missing data due to misspecification is a general concern that applies to alternative randomisation schemes and to covariates not involved in stratifying randomisation. While misspecification may have less impact in settings with smaller amounts of missing data or where covariates are less prognostic of missing data, we nonetheless encourage correct specification of analysis models to minimise the potential for biased treatment effect estimates in the presence of missing data.

In this article we predominantly focused on a limited set of direct adjustment approaches to accounting for stratification variables. Recent guidance from the Food and Drug Administration notes that Cochran-Mantel-Haenzsel methods are acceptable for estimating conditional treatment effects for binary outcomes,^19^ and in our literature review we observed use of this approach for handling continuous stratification variables. Yet the Cochran-Mantel-Haenzel test produced very similar treatment effect estimates to logistic regression with direct adjustment for randomisation categories, and so we do not recommend it for use with continuous stratification variables. Among alternatives to the evaluated approaches, we did not consider the use of inverse probability of treatment weighting, owing to its infrequent use and ability to only target marginal treatment effect measures.^30^ We also did not evaluate the strategy of adjusting simultaneously for randomisation categories and continuous values of the stratification variable, whether assuming a linear or non-linear relationship between continuous values and the outcome. In principle such a dual-adjustment approach should perform similarly to adjusting solely for continuous values for smooth covariate-outcome relationships, while offering advantages for step-function relationships. However, the method may be more prone to convergence issues in small samples, and in medical research we would rarely (if ever) encounter a true step-function relationship.

A limitation of this investigation is the restriction to settings involving 1:1 randomisation. Nominal SEs can be inappropriate under other allocation ratios when the covariate-outcome relationship is misspecified, and so robust SEs are recommended in such settings.^19^ However, we expect power losses due to misspecification and the potential for bias with missing data to similarly apply in trials with unequal allocation ratios. Another limitation is the brief consideration of settings involving an interaction between the stratification variable and treatment group. When estimating average treatment effects, guidance documents recommend omitting interaction terms from analysis models,^10,11,19^ albeit this may introduce bias in settings with missing outcome data due to model misspecification. Should it be deemed necessary to model interaction effects to avoid bias due to missing outcome data, the estimators of Bugni et al.^31^ or Ma et al.^21^ would be required, since the ordinary least squares variance estimator of the average treatment effect is no longer valid in models with interaction terms under stratified randomisation. Finally, we did not consider the use of inverse probability weighting to address missing data. The ANCOVA-type estimator in combination with inverse probability weighting is doubly robust, such that just one of the weighting and analysis models is required to be correctly specified for valid point estimation under a missing at random assumption.^14,32^ Yet this double robustness property does not seem to be a good reason for knowingly misspecifying the ANCOVA model (eg, by adjusting for randomisation categories of a continuous stratification variable)—particularly as the weighting model may be more challenging to specify—since this would sacrifice precision.

In conclusion, we recommend the use of flexible approaches such as two-term fractional polynomials or restricted cubic splines when adjusting for continuous stratification variables in the analysis of randomised trials. While stratified randomisation is extensively used, practical considerations for trial design and analysis are still being addressed. For example, recent work has demonstrated the importance of adjusting for stratification variables,^9^ links between the statistical properties of various estimators under both simple and stratified randomisation,^7,8^ methods for addressing misclassified stratification variables,^33^ and optimal approaches for creating stratification categories for skewed continuous prognostic variables.^34^ This paper adds to this growing literature by demonstrating the advantages of leaving continuous variables used for stratified randomisation as continuous during analysis.

## Supplementary Material

Data S1

Data S2

## Figures and Tables

**Table 1:** Type-I error rates in scenarios with a continuous outcome and complete data (nominal α= 0.05).^a^

Covariate–outcome relationship	Unadjusted	Adjust for categories	Linear	FP-2	Cubic splines
*f* (*X*) = *X*, moderate	0.038	0.046	0.048	0.052	0.048
*f* (*X*) = *X*, strong	0.022	0.047	0.046	0.048	0.048
*f* (*X*) = *e^X^*, moderate	0.039	0.049	0.048	0.048	0.047
*f* (*X*) = *e^X^*, strong	0.029	0.044	0.049	0.052	0.052
*f* (*X*) = *X*^2^, moderate	0.050	0.049	0.050	0.051	0.053
*f* (*X*) = *X*^2^, strong	0.050	0.050	0.050	0.055	0.052
*f* (*X*) = *X^strat^*, moderate	0.031	0.051	0.047	0.049	0.049
*f* (*X*) = *X^strat^*, strong	0.006	0.051	0.039	0.043	0.052

a The maximum Monte Carlo SE across all methods and scenarios was 0.0033.

**Table 2:** Empirical SE in scenarios with a continuous outcome, complete data, and treatment effect of 0.4.^a^

Covariate-outcome relationship	Unadjusted	Adjust for categories	Linear	FP-2	Cubic splines
*f* (*X*) = *X*, moderate	0.146	0.146	0.142	0.143	0.144
*f* (*X*) = *X*, strong	0.154	0.154	0.138	0.139	0.140
*f* (*X*) = *e^X^*, moderate	0.163	0.163	0.154	0.144	0.146
*f* (*X*) = *e^X^*, strong	0.210	0.210	0.181	0.143	0.149
*f* (*X*) = *X*^2^, moderate	0.157	0.157	0.157	0.140	0.141
*f* (*X*) = *X*^2^, strong	0.203	0.203	0.202	0.143	0.144
*f* (*X*) = *X^strat^*, moderate	0.142	0.141	0.146	0.146	0.145
*f* (*X*) = *X^strat^*, strong	0.141	0.141	0.158	0.156	0.151

a The maximum Monte Carlo standard error across all methods and scenarios was 0.0021.

**Table 3:** Power and relative bias in scenarios with a continuous outcome, an interaction between the stratification variable and treatment group, and an average treatment effect of 0.4.^a^

Measure	Covariate-outcome relationship	Outcome data	Unadjusted	Adjust for categories	Linear	FP-2	Cubic splines
Power (%)	*f* (*X*) = *X*, moderate	Complete	77.0	77.7	78.5	79.2	77.8
	*f* (*X*) = *X*, strong	Complete	68.8	71.4	73.5	74.9	72.5
	*f* (*X*) = *X*, moderate	Missing 30%	41.2	45.5	47.9	49.4	46.8
	*f* (*X*) = *X*, strong	Missing 30%	18.6	24.8	28.5	31.5	27.6
Relative bias (%)	*f* (*X*) = *X*, moderate	Complete	−0.3	−0.3	−0.2	1.0	−0.2
	*f* (*X*) = *X*, strong	Complete	−1.0	−1.0	−1.2	1.0	−1.4
	*f* (*X*) = *X*, moderate	Missing 30%	−22.9	−18.8	−16.5	−15.1	−17.0
	*f* (*X*) = *X*, strong	Missing 30%	−49.0	−40.7	−35.9	−33.2	−37.0

a The maximum Monte Carlo standard error across all methods and scenarios was 0.71% for power and 0.67% for relative bias.

**Table 4:** Treatment effect estimates for supplemental oxygen support for chronic lung disease from the DINO trial.

Method^a^	Odds ratio high vs standard DHA (95% CI)	*P*-value	Risk difference (95% CI)
Unadjusted	0.66 (0.44, 1.01)	0.057	−0.067 (−0.135, 0.001)
Adjust for categories	0.60 (0.38, 0.96)	0.034	−0.067 (−0.128, −0.006)
Cochran–Mantel–Haenszel^b^	0.61 (0.39, 0.97)	0.034	–
Linear	0.55 (0.34, 0.91)	0.020	−0.070 (−0.128, −0.012)
FP-2	0.55 (0.33, 0.92)	0.023	−0.067 (−0.124, −0.010)
Cubic splines	0.56 (0.34, 0.93)	0.025	−0.066 (−0.123, −0.009)

a Methods describe how birth weight was adjusted for in the analysis. Excluding the unadjusted analysis and the Cochran–Mantel-Haenszel test, all methods additionally adjusted for the other stratification factors hospital and sex.

b Stratified by birth weight category, hospital, and sex.

## Data Availability

The data that support the findings of this study are available on request from the corresponding author. The data are not publicly available due to privacy or ethical restrictions.
