# Supplementary material for: Categorisation of continuous covariates for stratified randomisation: How should we adjust?
Source: Stat Med. Author manuscript; Available in PMC 2024 Sep 5. (PMC7616414; doi:10.1002/sim.10060)
Supplement: Data S1 [file EMS198075-supplement-Data_S1.docx]

**Statistical code for continuous simulation study**

clear all

program define continuous_outcome

args seed numsim t_effect function

//random number seed, number simulations, treatment effect, type of covariate outcome relationship//

postfile results simnum m1_est m1_se m1_low m1_high m2_est m2_se m2_low m2_high m3_est m3_se m3_low m3_high m4_est m4_se m4_low m4_high m5_est m5_se m5_low m5_high using results, replace

set seed `seed'

forvalues i = 1/`numsim' {

* Create randomisation schedule with 2 strata and randomly permuted blocks of size 4

clear

set obs 400

egen stratum = seq(), from(0) to(1) block(200)

egen block_num = seq(), from(1) to(100) block(4)

bysort stratum block_num: egen trt = seq(), from(0) to(1) block(1)

gen random = uniform()

sort stratum block_num random

gen rand_order = _n

bysort stratum: egen stratum_order = seq(), from(1) to(200) block(1)

keep stratum trt stratum_order

save schedule, replace

* Generate baseline covariate and randomise observations

clear

set obs 200

gen study_id = _n

gen X = rnormal()

gen stratum = 0

replace stratum = 1 if X > 0

sort stratum study_id

bysort stratum: egen stratum_order = seq(), block(1)

merge 1:1 stratum stratum_order using schedule

keep if _merge == 3

drop _merge stratum_order

sort study_id // sort data by study_id to get back to original order of participants //

* Create function of the baseline covariate X

if "`function'" == "linear_moderate" {

gen X_transform = X

local beta2 = 0.39

}

if "`function'" == "linear_strong" {

gen X_transform = X

local beta2 = 0.78

}

if "`function'" == "exponential_moderate" {

gen X_transform = exp(X)

local beta2 = 0.30

}

if "`function'" == "exponential_strong " {

gen X_transform = exp(X)

local beta2 = 0.60

}

if "`function'" == "quadratic_moderate " {

gen X_transform = X*X

local beta2 = 0.37

}

if "`function'" == "quadratic_strong" {

gen X_transform = X*X

local beta2 = 0.74

}

if "`function'" == "step_moderate " {

gen X_transform = stratum

local beta2 = 1

}

if "`function'" == "step_strong " {

gen X_transform = stratum

local beta2 = 2

}

* Generate outcome data

gen residual = rnormal(0,1)

gen outcome = (`t_effect'*trt) + (`beta2'*X_transform) + residual

* Induce missing outcome data (comment out section for complete data)

generate prob_missing = exp(-1.12 + log(1.5)*trt + log(1.5)*X + log(1.5)*X*trt)/(1 + exp(-1.12 + log(1.5)*trt + log(1.5)*X + log(1.5)*X*trt))

generate u1 = runiform()

replace outcome = . if prob_missing > u1

* Unadjusted analysis – m1

quietly regress outcome trt

lincom trt

scalar m1_est = r(estimate)

scalar m1_se = r(se)

scalar m1_low = r(lb)

scalar m1_high = r(ub)

* Analysis adjusting for stratification categories – m2

quietly regress outcome trt stratum

lincom trt

scalar m2_est = r(estimate)

scalar m2_se = r(se)

scalar m2_low = r(lb)

scalar m2_high = r(ub)

* Analysis assuming linear relationship between X and outcome – m3

quietly regress outcome trt X

lincom trt

scalar m3_est = r(estimate)

scalar m3_se = r(se)

scalar m3_low = r(lb)

scalar m3_high = r(ub)

* Analysis using fractional polynomials – m4

quietly fp <X>, scale: regress outcome trt <X>

lincom trt

scalar m4_est = r(estimate)

scalar m4_se = r(se)

scalar m4_low = r(lb)

scalar m4_high = r(ub)

* Analysis using restricted cubic splines – m5

mkspline knot = X, cubic

quietly regress outcome trt knot1 knot2 knot3 knot4

lincom trt

scalar m5_est = r(estimate)

scalar m5_se = r(se)

scalar m5_low = r(lb)

scalar m5_high = r(ub)

* Store simulation results

post results (`i') (m1_est) (m1_se) (m1_low) (m1_high) (m2_est) (m2_se) (m2_low) (m2_high) (m3_est) (m3_se) (m3_low) (m3_high) (m4_est) (m4_se) (m4_low) (m4_high) (m5_est) (m5_se) (m5_low) (m5_high)

}

postclose results

end

* Example call to simulation program

continuous_outcome 1893 5000 0.4 "linear_moderate" //simulation results saved in file “results.dta”//
