# Supplementary material for: Categorisation of continuous covariates for stratified randomisation: How should we adjust?
Source: Stat Med. Author manuscript; Available in PMC 2024 Sep 5. (PMC7616414; doi:10.1002/sim.10060)
Supplement: Data S2 [file EMS198075-supplement-Data_S2.docx]

**SUPPLEMENTARY MATERIALS**

**Eligible articles identified in systematic review**

1. Al-Samkari H, Galactéros F, Glenthøj A, et al. Mitapivat versus Placebo for Pyruvate Kinase Deficiency. *N Engl J Med*. 2022;386(15):1432-1442.

2. Alhazzani W, Parhar KKS, Weatherald J, et al. Effect of Awake Prone Positioning on Endotracheal Intubation in Patients With COVID-19 and Acute Respiratory Failure: A Randomized Clinical Trial. *Jama*. 2022;327(21):2104-2113.

3. Ansell SM, Radford J, Connors JM, et al. Overall Survival with Brentuximab Vedotin in Stage III or IV Hodgkin's Lymphoma. *N Engl J Med*. 2022;387(4):310-320.

4. Basch E, Schrag D, Henson S, et al. Effect of Electronic Symptom Monitoring on Patient-Reported Outcomes Among Patients With Metastatic Cancer: A Randomized Clinical Trial. *Jama*. 2022;327(24):2413-2422.

5. Bearne LM, Volkmer B, Peacock J, et al. Effect of a Home-Based, Walking Exercise Behavior Change Intervention vs Usual Care on Walking in Adults With Peripheral Artery Disease: The MOSAIC Randomized Clinical Trial. *Jama*. 2022;327(14):1344-1355.

6. Bernabei R, Landi F, Calvani R, et al. Multicomponent intervention to prevent mobility disability in frail older adults: randomised controlled trial (SPRINTT project). *Bmj*. 2022;377:e068788.

7. Bisson JI, Ariti C, Cullen K, et al. Guided, internet based, cognitive behavioural therapy for post-traumatic stress disorder: pragmatic, multicentre, randomised controlled non-inferiority trial (RAPID). *Bmj*. 2022;377:e069405.

8. Bösel J, Niesen WD, Salih F, et al. Effect of Early vs Standard Approach to Tracheostomy on Functional Outcome at 6 Months Among Patients With Severe Stroke Receiving Mechanical Ventilation: The SETPOINT2 Randomized Clinical Trial. *Jama*. 2022;327(19):1899-1909.

9. Bradbury CA, Lawler PR, Stanworth SJ, et al. Effect of Antiplatelet Therapy on Survival and Organ Support-Free Days in Critically Ill Patients With COVID-19: A Randomized Clinical Trial. *Jama*. 2022;327(13):1247-1259.

10. Catto JWF, Khetrapal P, Ricciardi F, et al. Effect of Robot-Assisted Radical Cystectomy With Intracorporeal Urinary Diversion vs Open Radical Cystectomy on 90-Day Morbidity and Mortality Among Patients With Bladder Cancer: A Randomized Clinical Trial. *Jama*. 2022;327(21):2092-2103.

11. Chappell LC, Tucker KL, Galal U, et al. Effect of Self-monitoring of Blood Pressure on Blood Pressure Control in Pregnant Individuals With Chronic or Gestational Hypertension: The BUMP 2 Randomized Clinical Trial. *Jama*. 2022;327(17):1666-1678.

12. Cortes J, Rugo HS, Cescon DW, et al. Pembrolizumab plus Chemotherapy in Advanced Triple-Negative Breast Cancer. *N Engl J Med*. 2022;387(3):217-226.

13. Cox LS, Nollen NL, Mayo MS, et al. Effect of Varenicline Added to Counseling on Smoking Cessation Among African American Daily Smokers: The Kick It at Swope IV Randomized Clinical Trial. *Jama*. 2022;327(22):2201-2209.

14. Creech CB, Anderson E, Berthaud V, et al. Evaluation of mRNA-1273 Covid-19 Vaccine in Children 6 to 11 Years of Age. *N Engl J Med*. 2022;386(21):2011-2023.

15. D'Haens G, Panaccione R, Baert F, et al. Risankizumab as induction therapy for Crohn's disease: results from the phase 3 ADVANCE and MOTIVATE induction trials. *Lancet*. 2022;399(10340):2015-2030.

16. Dai L, Gao L, Tao L, et al. Efficacy and Safety of the RBD-Dimer-Based Covid-19 Vaccine ZF2001 in Adults. *N Engl J Med*. 2022;386(22):2097-2111.

17. Danese S, Vermeire S, Zhou W, et al. Upadacitinib as induction and maintenance therapy for moderately to severely active ulcerative colitis: results from three phase 3, multicentre, double-blind, randomised trials. *Lancet*. 2022;399(10341):2113-2128.

18. Delany-Moretlwe S, Hughes JP, Bock P, et al. Cabotegravir for the prevention of HIV-1 in women: results from HPTN 084, a phase 3, randomised clinical trial. *Lancet*. 2022;399(10337):1779-1789.

19. Delgado-Lista J, Alcala-Diaz JF, Torres-Peña JD, et al. Long-term secondary prevention of cardiovascular disease with a Mediterranean diet and a low-fat diet (CORDIOPREV): a randomised controlled trial. *Lancet*. 2022;399(10338):1876-1885.

20. Devereaux PJ, Marcucci M, Painter TW, et al. Tranexamic Acid in Patients Undergoing Noncardiac Surgery. *N Engl J Med*. 2022;386(21):1986-1997.

21. Eckburg PB, Muir L, Critchley IA, et al. Oral Tebipenem Pivoxil Hydrobromide in Complicated Urinary Tract Infection. *N Engl J Med*. 2022;386(14):1327-1338.

22. Edqvist M, Dahlen HG, Häggsgård C, et al. The effect of two midwives during the second stage of labour to reduce severe perineal trauma (Oneplus): a multicentre, randomised controlled trial in Sweden. *Lancet*. 2022;399(10331):1242-1253.

23. Ezekowitz JA, Colin-Ramirez E, Ross H, et al. Reduction of dietary sodium to less than 100 mmol in heart failure (SODIUM-HF): an international, open-label, randomised, controlled trial. *Lancet*. 2022;399(10333):1391-1400.

24. Ferrante M, Panaccione R, Baert F, et al. Risankizumab as maintenance therapy for moderately to severely active Crohn's disease: results from the multicentre, randomised, double-blind, placebo-controlled, withdrawal phase 3 FORTIFY maintenance trial. *Lancet*. 2022;399(10340):2031-2046.

25. Fischer U, Kaesmacher J, Strbian D, et al. Thrombectomy alone versus intravenous alteplase plus thrombectomy in patients with stroke: an open-label, blinded-outcome, randomised non-inferiority trial. *Lancet*. 2022;400(10346):104-115.

26. Fizazi K, Foulon S, Carles J, et al. Abiraterone plus prednisone added to androgen deprivation therapy and docetaxel in de novo metastatic castration-sensitive prostate cancer (PEACE-1): a multicentre, open-label, randomised, phase 3 study with a 2 × 2 factorial design. *Lancet*. 2022;399(10336):1695-1707.

27. Forde PM, Spicer J, Lu S, et al. Neoadjuvant Nivolumab plus Chemotherapy in Resectable Lung Cancer. *N Engl J Med*. 2022;386(21):1973-1985.

28. Giudice LC, As-Sanie S, Arjona Ferreira JC, et al. Once daily oral relugolix combination therapy versus placebo in patients with endometriosis-associated pain: two replicate phase 3, randomised, double-blind, studies (SPIRIT 1 and 2). *Lancet*. 2022;399(10343):2267-2279.

29. Goodwin PJ, Chen BE, Gelmon KA, et al. Effect of Metformin vs Placebo on Invasive Disease-Free Survival in Patients With Breast Cancer: The MA.32 Randomized Clinical Trial. *Jama*. 2022;327(20):1963-1973.

30. Guglieri M, Bushby K, McDermott MP, et al. Effect of Different Corticosteroid Dosing Regimens on Clinical Outcomes in Boys With Duchenne Muscular Dystrophy: A Randomized Clinical Trial. *Jama*. 2022;327(15):1456-1468.

31. Gupta A, Gonzalez-Rojas Y, Juarez E, et al. Effect of Sotrovimab on Hospitalization or Death Among High-risk Patients With Mild to Moderate COVID-19: A Randomized Clinical Trial. *Jama*. 2022;327(13):1236-1246.

32. Hager KJ, Pérez Marc G, Gobeil P, et al. Efficacy and Safety of a Recombinant Plant-Based Adjuvanted Covid-19 Vaccine. *N Engl J Med*. 2022;386(22):2084-2096.

33. Hammond J, Leister-Tebbe H, Gardner A, et al. Oral Nirmatrelvir for High-Risk, Nonhospitalized Adults with Covid-19. *N Engl J Med*. 2022;386(15):1397-1408.

34. Hodgson KA, Owen LS, Kamlin COF, et al. Nasal High-Flow Therapy during Neonatal Endotracheal Intubation. *N Engl J Med*. 2022;386(17):1627-1637.

35. Israel E, Cardet JC, Carroll JK, et al. Reliever-Triggered Inhaled Glucocorticoid in Black and Latinx Adults with Asthma. *N Engl J Med*. 2022;386(16):1505-1518.

36. Jastreboff AM, Aronne LJ, Ahmad NN, et al. Tirzepatide Once Weekly for the Treatment of Obesity. *N Engl J Med*. 2022;387(3):205-216.

37. Kamdar M, Solomon SR, Arnason J, et al. Lisocabtagene maraleucel versus standard of care with salvage chemotherapy followed by autologous stem cell transplantation as second-line treatment in patients with relapsed or refractory large B-cell lymphoma (TRANSFORM): results from an interim analysis of an open-label, randomised, phase 3 trial. *Lancet*. 2022;399(10343):2294-2308.

38. Kawahara T, Suzuki G, Mizuno S, et al. Effect of active vitamin D treatment on development of type 2 diabetes: DPVD randomised controlled trial in Japanese population. *Bmj*. 2022;377:e066222.

39. Khobragade A, Bhate S, Ramaiah V, et al. Efficacy, safety, and immunogenicity of the DNA SARS-CoV-2 vaccine (ZyCoV-D): the interim efficacy results of a phase 3, randomised, double-blind, placebo-controlled study in India. *Lancet*. 2022;399(10332):1313-1321.

40. King B, Ohyama M, Kwon O, et al. Two Phase 3 Trials of Baricitinib for Alopecia Areata. *N Engl J Med*. 2022;386(18):1687-1699.

41. Kotwal S, Cass A, Coggan S, et al. Multifaceted intervention to reduce haemodialysis catheter related bloodstream infections: REDUCCTION stepped wedge, cluster randomised trial. *Bmj*. 2022;377:e069634.

42. Krijbolder DI, Verstappen M, van Dijk BT, et al. Intervention with methotrexate in patients with arthralgia at risk of rheumatoid arthritis to reduce the development of persistent arthritis and its disease burden (TREAT EARLIER): a randomised, double-blind, placebo-controlled, proof-of-concept trial. *Lancet*. 2022;400(10348):283-294.

43. Lamontagne F, Masse MH, Menard J, et al. Intravenous Vitamin C in Adults with Sepsis in the Intensive Care Unit. *N Engl J Med*. 2022;386(25):2387-2398.

44. LeBoff MS, Chou SH, Ratliff KA, et al. Supplemental Vitamin D and Incident Fractures in Midlife and Older Adults. *N Engl J Med*. 2022;387(4):299-309.

45. Levin MJ, Ustianowski A, De Wit S, et al. Intramuscular AZD7442 (Tixagevimab-Cilgavimab) for Prevention of Covid-19. *N Engl J Med*. 2022;386(23):2188-2200.

46. Lewis GD, Voors AA, Cohen-Solal A, et al. Effect of Omecamtiv Mecarbil on Exercise Capacity in Chronic Heart Failure With Reduced Ejection Fraction: The METEORIC-HF Randomized Clinical Trial. *Jama*. 2022;328(3):259-269.

47. Liu D, Huang Y, Huang C, et al. Calorie Restriction with or without Time-Restricted Eating in Weight Loss. *N Engl J Med*. 2022;386(16):1495-1504.

48. Lu Z, Wang J, Shu Y, et al. Sintilimab versus placebo in combination with chemotherapy as first line treatment for locally advanced or metastatic oesophageal squamous cell carcinoma (ORIENT-15): multicentre, randomised, double blind, phase 3 trial. *Bmj*. 2022;377:e068714.

49. Luke JJ, Rutkowski P, Queirolo P, et al. Pembrolizumab versus placebo as adjuvant therapy in completely resected stage IIB or IIC melanoma (KEYNOTE-716): a randomised, double-blind, phase 3 trial. *Lancet*. 2022;399(10336):1718-1729.

50. Lv J, Wong MG, Hladunewich MA, et al. Effect of Oral Methylprednisolone on Decline in Kidney Function or Kidney Failure in Patients With IgA Nephropathy: The TESTING Randomized Clinical Trial. *Jama*. 2022;327(19):1888-1898.

51. Mahfoud F, Kandzari DE, Kario K, et al. Long-term efficacy and safety of renal denervation in the presence of antihypertensive drugs (SPYRAL HTN-ON MED): a randomised, sham-controlled trial. *Lancet*. 2022;399(10333):1401-1410.

52. Marrouche NF, Wazni O, McGann C, et al. Effect of MRI-Guided Fibrosis Ablation vs Conventional Catheter Ablation on Atrial Arrhythmia Recurrence in Patients With Persistent Atrial Fibrillation: The DECAAF II Randomized Clinical Trial. *Jama*. 2022;327(23):2296-2305.

53. Maurovich-Horvat P, Bosserdt M, Kofoed KF, et al. CT or Invasive Coronary Angiography in Stable Chest Pain. *N Engl J Med*. 2022;386(17):1591-1602.

54. McConnell MA, Rokicki S, Ayers S, et al. Effect of an Intensive Nurse Home Visiting Program on Adverse Birth Outcomes in a Medicaid-Eligible Population: A Randomized Clinical Trial. *Jama*. 2022;328(1):27-37.

55. Meersch M, Weiss R, Küllmar M, et al. Effect of Intraoperative Handovers of Anesthesia Care on Mortality, Readmission, or Postoperative Complications Among Adults: The HandiCAP Randomized Clinical Trial. *Jama*. 2022;327(24):2403-2412.

56. Melnick ER, Nath B, Dziura JD, et al. User centered clinical decision support to implement initiation of buprenorphine for opioid use disorder in the emergency department: EMBED pragmatic cluster randomized controlled trial. *Bmj*. 2022;377:e069271.

57. Menon BK, Buck BH, Singh N, et al. Intravenous tenecteplase compared with alteplase for acute ischaemic stroke in Canada (AcT): a pragmatic, multicentre, open-label, registry-linked, randomised, controlled, non-inferiority trial. *Lancet*. 2022;400(10347):161-169.

58. Metcalfe A, Parsons H, Parsons N, et al. Subacromial balloon spacer for irreparable rotator cuff tears of the shoulder (START:REACTS): a group-sequential, double-blind, multicentre randomised controlled trial. *Lancet*. 2022;399(10339):1954-1963.

59. Meyhoff TS, Hjortrup PB, Wetterslev J, et al. Restriction of Intravenous Fluid in ICU Patients with Septic Shock. *N Engl J Med*. 2022;386(26):2459-2470.

60. Mitchell PJ, Yan B, Churilov L, et al. Endovascular thrombectomy versus standard bridging thrombolytic with endovascular thrombectomy within 4·5 h of stroke onset: an open-label, blinded-endpoint, randomised non-inferiority trial. *Lancet*. 2022;400(10346):116-125.

61. Modi S, Jacot W, Yamashita T, et al. Trastuzumab Deruxtecan in Previously Treated HER2-Low Advanced Breast Cancer. *N Engl J Med*. 2022;387(1):9-20.

62. Montesinos P, Recher C, Vives S, et al. Ivosidenib and Azacitidine in IDH1-Mutated Acute Myeloid Leukemia. *N Engl J Med*. 2022;386(16):1519-1531.

63. Moreira ED, Jr., Kitchin N, Xu X, et al. Safety and Efficacy of a Third Dose of BNT162b2 Covid-19 Vaccine. *N Engl J Med*. 2022;386(20):1910-1921.

64. Mosha JF, Kulkarni MA, Lukole E, et al. Effectiveness and cost-effectiveness against malaria of three types of dual-active-ingredient long-lasting insecticidal nets (LLINs) compared with pyrethroid-only LLINs in Tanzania: a four-arm, cluster-randomised trial. *Lancet*. 2022;399(10331):1227-1241.

65. Myhrvold SB, Brouwer EF, Andresen TKM, et al. Nonoperative or Surgical Treatment of Acute Achilles' Tendon Rupture. *N Engl J Med*. 2022;386(15):1409-1420.

66. Nguyen TT, Chiu CH, Lin CY, et al. Efficacy, safety, and immunogenicity of an inactivated, adjuvanted enterovirus 71 vaccine in infants and children: a multiregion, double-blind, randomised, placebo-controlled, phase 3 trial. *Lancet*. 2022;399(10336):1708-1717.

67. Oslin DW, Lynch KG, Shih MC, et al. Effect of Pharmacogenomic Testing for Drug-Gene Interactions on Medication Selection and Remission of Symptoms in Major Depressive Disorder: The PRIME Care Randomized Clinical Trial. *Jama*. 2022;328(2):151-161.

68. Palefsky JM, Lee JY, Jay N, et al. Treatment of Anal High-Grade Squamous Intraepithelial Lesions to Prevent Anal Cancer. *N Engl J Med*. 2022;386(24):2273-2282.

69. Papi A, Chipps BE, Beasley R, et al. Albuterol-Budesonide Fixed-Dose Combination Rescue Inhaler for Asthma. *N Engl J Med*. 2022;386(22):2071-2083.

70. Paskins Z, Bromley K, Lewis M, et al. Clinical effectiveness of one ultrasound guided intra-articular corticosteroid and local anaesthetic injection in addition to advice and education for hip osteoarthritis (HIT trial): single blind, parallel group, three arm, randomised controlled trial. *Bmj*. 2022;377:e068446.

71. Pérez de la Ossa N, Abilleira S, Jovin TG, et al. Effect of Direct Transportation to Thrombectomy-Capable Center vs Local Stroke Center on Neurological Outcomes in Patients With Suspected Large-Vessel Occlusion Stroke in Nonurban Areas: The RACECAT Randomized Clinical Trial. *Jama*. 2022;327(18):1782-1794.

72. Perry DC, Achten J, Knight R, et al. Immobilisation of torus fractures of the wrist in children (FORCE): a randomised controlled equivalence trial in the UK. *Lancet*. 2022;400(10345):39-47.

73. Pesonen E, Vlasov H, Suojaranta R, et al. Effect of 4% Albumin Solution vs Ringer Acetate on Major Adverse Events in Patients Undergoing Cardiac Surgery With Cardiopulmonary Bypass: A Randomized Clinical Trial. *Jama*. 2022;328(3):251-258.

74. Piccini JP, Caso V, Connolly SJ, et al. Safety of the oral factor XIa inhibitor asundexian compared with apixaban in patients with atrial fibrillation (PACIFIC-AF): a multicentre, randomised, double-blind, double-dummy, dose-finding phase 2 study. *Lancet*. 2022;399(10333):1383-1390.

75. Pollack A, Karrison TG, Balogh AG, et al. The addition of androgen deprivation therapy and pelvic lymph node treatment to prostate bed salvage radiotherapy (NRG Oncology/RTOG 0534 SPPORT): an international, multicentre, randomised phase 3 trial. *Lancet*. 2022;399(10338):1886-1901.

76. Räber L, Ueki Y, Otsuka T, et al. Effect of Alirocumab Added to High-Intensity Statin Therapy on Coronary Atherosclerosis in Patients With Acute Myocardial Infarction: The PACMAN-AMI Randomized Clinical Trial. *Jama*. 2022;327(18):1771-1781.

77. Ramnarayan P, Richards-Belle A, Drikite L, et al. Effect of High-Flow Nasal Cannula Therapy vs Continuous Positive Airway Pressure Following Extubation on Liberation From Respiratory Support in Critically Ill Children: A Randomized Clinical Trial. *Jama*. 2022;327(16):1555-1565.

78. Ramnarayan P, Richards-Belle A, Drikite L, et al. Effect of High-Flow Nasal Cannula Therapy vs Continuous Positive Airway Pressure Therapy on Liberation From Respiratory Support in Acutely Ill Children Admitted to Pediatric Critical Care Units: A Randomized Clinical Trial. *Jama*. 2022;328(2):162-172.

79. Reich K, Thyssen JP, Blauvelt A, et al. Efficacy and safety of abrocitinib versus dupilumab in adults with moderate-to-severe atopic dermatitis: a randomised, double-blind, multicentre phase 3 trial. *Lancet*. 2022;400(10348):273-282.

80. Reis G, Silva E, Silva DCM, et al. Effect of Early Treatment with Ivermectin among Patients with Covid-19. *N Engl J Med*. 2022;386(18):1721-1731.

81. Richardson PG, Jacobus SJ, Weller EA, et al. Triplet Therapy, Transplantation, and Maintenance until Progression in Myeloma. *N Engl J Med*. 2022;387(2):132-147.

82. Richeldi L, Azuma A, Cottin V, et al. Trial of a Preferential Phosphodiesterase 4B Inhibitor for Idiopathic Pulmonary Fibrosis. *N Engl J Med*. 2022;386(23):2178-2187.

83. Russell DW, Casey JD, Gibbs KW, et al. Effect of Fluid Bolus Administration on Cardiovascular Collapse Among Critically Ill Patients Undergoing Tracheal Intubation: A Randomized Clinical Trial. *Jama*. 2022;328(3):270-279.

84. Saji H, Okada M, Tsuboi M, et al. Segmentectomy versus lobectomy in small-sized peripheral non-small-cell lung cancer (JCOG0802/WJOG4607L): a multicentre, open-label, phase 3, randomised, controlled, non-inferiority trial. *Lancet*. 2022;399(10335):1607-1617.

85. Sands BE, Irving PM, Hoops T, et al. Ustekinumab versus adalimumab for induction and maintenance therapy in biologic-naive patients with moderately to severely active Crohn's disease: a multicentre, randomised, double-blind, parallel-group, phase 3b trial. *Lancet*. 2022;399(10342):2200-2211.

86. Schlapbach LJ, Gibbons KS, Horton SB, et al. Effect of Nitric Oxide via Cardiopulmonary Bypass on Ventilator-Free Days in Young Children Undergoing Congenital Heart Disease Surgery: The NITRIC Randomized Clinical Trial. *Jama*. 2022;328(1):38-47.

87. Schmoele-Thoma B, Zareba AM, Jiang Q, et al. Vaccine Efficacy in Adults in a Respiratory Syncytial Virus Challenge Study. *N Engl J Med*. 2022;386(25):2377-2386.

88. Segal-Maurer S, DeJesus E, Stellbrink HJ, et al. Capsid Inhibition with Lenacapavir in Multidrug-Resistant HIV-1 Infection. *N Engl J Med*. 2022;386(19):1793-1803.

89. Sessler DI, Pei L, Li K, et al. Aggressive intraoperative warming versus routine thermal management during non-cardiac surgery (PROTECT): a multicentre, parallel group, superiority trial. *Lancet*. 2022;399(10337):1799-1808.

90. Shi J, Zhou C, Pan W, et al. Effect of High- vs Low-Dose Tranexamic Acid Infusion on Need for Red Blood Cell Transfusion and Adverse Events in Patients Undergoing Cardiac Surgery: The OPTIMAL Randomized Clinical Trial. *Jama*. 2022;328(4):336-347.

91. Simões EAF, Center KJ, Tita ATN, et al. Prefusion F Protein-Based Respiratory Syncytial Virus Immunization in Pregnancy. *N Engl J Med*. 2022;386(17):1615-1626.

92. Skjerven HO, Lie A, Vettukattil R, et al. Early food intervention and skin emollients to prevent food allergy in young children (PreventADALL): a factorial, multicentre, cluster-randomised trial. *Lancet*. 2022;399(10344):2398-2411.

93. Smits FJ, Henry AC, Besselink MG, et al. Algorithm-based care versus usual care for the early recognition and management of complications after pancreatic resection in the Netherlands: an open-label, nationwide, stepped-wedge cluster-randomised trial. *Lancet*. 2022;399(10338):1867-1875.

94. Stahl A, Sukgen EA, Wu WC, et al. Effect of Intravitreal Aflibercept vs Laser Photocoagulation on Treatment Success of Retinopathy of Prematurity: The FIREFLEYE Randomized Clinical Trial. *Jama*. 2022;328(4):348-359.

95. Sullivan DJ, Gebo KA, Shoham S, et al. Early Outpatient Treatment for Covid-19 with Convalescent Plasma. *N Engl J Med*. 2022;386(18):1700-1711.

96. Sun Y, Mu J, Wang DW, et al. A village doctor-led multifaceted intervention for blood pressure control in rural China: an open, cluster randomised trial. *Lancet*. 2022;399(10339):1964-1975.

97. Tie J, Cohen JD, Lahouel K, et al. Circulating Tumor DNA Analysis Guiding Adjuvant Therapy in Stage II Colon Cancer. *N Engl J Med*. 2022;386(24):2261-2272.

98. Tita AT, Szychowski JM, Boggess K, et al. Treatment for Mild Chronic Hypertension during Pregnancy. *N Engl J Med*. 2022;386(19):1781-1792.

99. Toff WD, Hildick-Smith D, Kovac J, et al. Effect of Transcatheter Aortic Valve Implantation vs Surgical Aortic Valve Replacement on All-Cause Mortality in Patients With Aortic Stenosis: A Randomized Clinical Trial. *Jama*. 2022;327(19):1875-1887.

100. Trutnovsky G, Reich O, Joura EA, et al. Topical imiquimod versus surgery for vulvar intraepithelial neoplasia: a multicentre, randomised, phase 3, non-inferiority trial. *Lancet*. 2022;399(10337):1790-1798.

101. Tucker KL, Mort S, Yu LM, et al. Effect of Self-monitoring of Blood Pressure on Diagnosis of Hypertension During Higher-Risk Pregnancy: The BUMP 1 Randomized Clinical Trial. *Jama*. 2022;327(17):1656-1665.

102. Wang ML, Jurczak W, Jerkeman M, et al. Ibrutinib plus Bendamustine and Rituximab in Untreated Mantle-Cell Lymphoma. *N Engl J Med*. 2022;386(26):2482-2494.

103. WHO Solidarity Trial Consortium. Remdesivir and three other drugs for hospitalised patients with COVID-19: final results of the WHO Solidarity randomised trial and updated meta-analyses. *Lancet*. 2022;399(10339):1941-1953.

104. Wu YW, Comstock BA, Gonzalez FF, et al. Trial of Erythropoietin for Hypoxic-Ischemic Encephalopathy in Newborns. *N Engl J Med*. 2022;387(2):148-159.

105. Yoshimura S, Sakai N, Yamagami H, et al. Endovascular Therapy for Acute Stroke with a Large Ischemic Region. *N Engl J Med*. 2022;386(14):1303-1313.

**Additional simulation results for continuous outcome data**

**Figure S1.** Percentage bias in sensitivity analysis scenarios involving simple randomisation, a continuous outcome, 30% missing outcome data, strong covariate–outcome relationship, and treatment effect of 0.4. The maximum Monte Carlo standard error across all methods and scenarios was 0.87%.

**Simulation methods for binary outcome data**

For each simulation scenario involving binary outcome data, 5,000 datasets of 800 observations (participants) were generated. As in the continuous simulation study, the $i$^th^ participant in each dataset was randomised to treatment group $T_{i}$, stratifying on $X_{i}^{strat}$ (derived from $X_{i}\sim N\left( 0,1 \right)$) using randomly permuted blocks of size 4. Binary outcome data were generated from the model $log(\frac{p}{1-p})=\beta_{0}+\beta_{1}T_{i}+\beta_{2}f\left( X_{i} \right)$, with $p$ the probability of the binary outcome and $f\left( X \right)=X$, $e^{X}$, $X^{2}$, or $X^{strat}$ as for the continuous outcome setting. The intercept term $\beta_{0}$ was chosen to produce an outcome prevalence of 50% in the control arm, while $\beta_{1}$ was set to log(1.5) such that the conditional odds ratio for the effect of treatment was 1.5. The treatment effect and sample size were chosen based on power considerations (400 observations per group provides approximately 80% power to detect an increase from 50% in the control arm to 60% in the intervention arm (odds ratio = 1.5), alpha = 0.05 two-tailed). The covariate effect $\beta_{2}$was chosen so that a change from the 10^th^ to the 90^th^ percentile in $f\left( X \right)$ increased the log odds of the outcome by $\frac{\pi}{\sqrt{3}}$ or $\frac{2\pi}{\sqrt{3}}$ (with $\frac{\pi}{\sqrt{3}}$ the standard deviation of a standard logistic distribution), again denoted moderate and strong covariate-outcome relationships, respectively. In addition to complete data settings, simulations were repeated where outcome values were randomly set to missing according to the model $\text{logit} P\left( Y_{i} \text{missing} \right)=\gamma+\log\left( 1.5 \right)T_{i}+\log\left( 1.5 \right)X_{i}+\log\left( 1.5 \right)X_{i}T_{i}$, with the intercept term $\gamma$ set to produce 30% missing outcome data overall.

Generated datasets were analysed using logistic regression, with (1) no adjustment; (2) adjustment for the randomisation categories $X^{strat}$; (3) adjustment for continuous $X$ assuming a linear relationship with the log-odds of the outcome; (4) adjustment for continuous $X$ using two-term fractional polynomials; (5) adjustment for continuous $X$ using restricted cubic splines with 5 knots placed using the standard percentile method; and (6) the Cochran–Mantel–Haenszel (CMH) test with stratification for the randomisation categories. The unadjusted analysis was included for comparison, albeit such an analysis targets a different estimand due to non-collapsibility of the odds ratio, while the CMH test was added due to its observed use in the literature review. Of interest was the performance of the methods in estimating the treatment effect $\beta_{1}$ expressed as a log-odds ratio, with performance described according to the percentage difference in estimated treatment effects from the true log-odds ratio, coverage of 95% confidence intervals (or type-I error rate for $\beta_{1}$= 0), empirical standard error and power (for $\beta_{1}$= log(1.5)).

Given increasing interest in marginal treatment effects for binary outcome data, we also considered estimation of the risk difference using the logistic models described above combined with standardisation applied using the margins command in Stata (with the delta method used to calculate standard errors). In settings with missing data, we used the “esample” option in Stata to estimate the treatment effect in all randomised participants rather than only those with complete outcome data, as we expected the risk difference to differ between participants with or without missing data due to differences in the distribution of $X$ between these groups. Estimates of the risk difference were compared against the “true” risk difference, obtained by comparing proportions between randomised groups in a single large, simulated dataset (50,000,000 observations chosen according to computational constraints). While the conditional odds ratio was fixed at 1.5, the true risk difference varied between 0.073 and 0.09 across scenarios involving linear, exponential, and quadratic covariate–outcome relationships, and between 0.049 and 0.083 across scenarios involving the step function relationship.

**Simulation results for binary outcome data**

When the treatment effect $\beta_{1}$ was set to 0, all methods were unbiased (results not shown) and exhibited appropriate type-I error rates for the conditional odds ratio (Table S1), except for the unadjusted analysis which produced type-I error rates below the nominal 0.05 level for linear, exponential and step function covariate–outcome relationships. Similar results were observed for the risk difference (results not shown).

**Table S1.** Type-I error rate for the log odds ratio in scenarios with a binary outcome and complete data*

| Covariate–outcome relationship | Unadjusted | Adjust for categories | Linear | FP-2 | Cubic splines | CMH |
| --- | --- | --- | --- | --- | --- | --- |
| $f\left( X \right)=X$, moderate | 0.046 | 0.052 | 0.051 | 0.052 | 0.051 | 0.052 |
| $f\left( X \right)=X$, strong | 0.034 | 0.056 | 0.058 | 0.058 | 0.058 | 0.056 |
| $f\left( X \right)=e^{X}$, moderate | 0.048 | 0.052 | 0.051 | 0.051 | 0.050 | 0.052 |
| $f\left( X \right)=e^{X}$, strong | 0.033 | 0.050 | 0.051 | 0.053 | 0.052 | 0.050 |
| $f\left( X \right)=X^{2}$, moderate | 0.046 | 0.046 | 0.045 | 0.043 | 0.042 | 0.046 |
| $f\left( X \right)=X^{2}$, strong | 0.050 | 0.050 | 0.049 | 0.051 | 0.050 | 0.050 |
| $f\left( X \right)=X^{strat}$, moderate | 0.034 | 0.052 | 0.048 | 0.049 | 0.051 | 0.052 |
| $f\left( X \right)=X^{strat}$, strong | 0.004 | 0.051 | 0.036 | 0.038 | 0.047 | 0.051 |

* The maximum Monte Carlo standard error across all methods and scenarios was 0.0033.

When $\beta_{1}$ was set to log(1.5) and interest concerned the conditional odds ratio, misspecification of the covariate–outcome relationship in an adjusted analysis was associated with attenuation of the treatment effect estimate (Figure S2) and reductions in statistical power (Figure S3). Unadjusted analysis also produced noticeably attenuated treatment effect estimates (due to non-collapsibility) with reduced power across all settings. The degree of attenuation and loss of power associated with misspecification was more pronounced in settings involving strong covariate–outcome relationships. As the sample size and conditional effect of treatment $\beta_{1}$ were fixed, note the power of appropriately specified approaches varied across simulation scenarios according to the functional form and strength of the covariate–outcome relationship. When standardisation was used following logistic regression to estimate the risk difference, all analysis methods produced unbiased treatment effect estimates, with only the unadjusted analysis exhibiting any problems with coverage (over-coverage for $f\left( X \right)=X$, $f\left( X \right)=e^{X}$ and $f\left( X \right)=X^{strat}$; results not shown). As in the continuous simulation study, empirical standard errors for the risk difference among methods that adjusted for the stratification variable in some form were lowest for approaches that correctly specified the covariate–outcome relationship (Table S2). Differences between analysis approaches in statistical power for the risk difference very closely resembled those displayed in Figure S3 for the conditional odds ratio (results not shown).

**Figure S2.** Relative difference in log odds ratio estimate from the true conditional effect of log(1.5) in scenarios with a binary outcome and complete data. The maximum Monte Carlo standard error across all methods and scenarios was 0.73%.

**Figure S3.** Power in scenarios with a binary outcome, complete data, conditional treatment effect of log(1.5), and the conditional odds ratio as the target for inference. The maximum Monte Carlo standard error across all methods and scenarios was 0.70%.

**Table S2.** Empirical standard error for the risk difference in scenarios with a binary outcome, complete data, and treatment effect of log(1.5)*

| Covariate–outcome relationship | Unadjusted | Adjust for categories | Linear | FP-2 | Cubic splines |
| --- | --- | --- | --- | --- | --- |
| $f\left( X \right)=X$, moderate | 0.0336 | 0.0366 | 0.0331 | 0.0331 | 0.0331 |
| $f\left( X \right)=X$, strong | 0.0318 | 0.0318 | 0.0302 | 0.0302 | 0.0303 |
| $f\left( X \right)=e^{X}$, moderate | 0.0343 | 0.0343 | 0.0338 | 0.0336 | 0.0336 |
| $f\left( X \right)=e^{X}$, strong | 0.0326 | 0.0326 | 0.0318 | 0.0314 | 0.0314 |
| $f\left( X \right)=X^{2}$, moderate | 0.0348 | 0.0348 | 0.0348 | 0.0329 | 0.0329 |
| $f\left( X \right)=X^{2}$, strong | 0.0351 | 0.0351 | 0.0351 | 0.0312 | 0.0312 |
| $f\left( X \right)=X^{strat}$, moderate | 0.0320 | 0.0319 | 0.0327 | 0.0327 | 0.0325 |
| $f\left( X \right)=X^{strat}$, strong | 0.0250 | 0.0249 | 0.0269 | 0.0268 | 0.0262 |

* The maximum Monte Carlo standard error across all methods and scenarios was 0.00035.

While misspecification of the covariate–outcome relationship did not lead to biased estimates of the risk difference with complete data, the same did not hold with covariate-dependent missing data in the outcome. Figure S4 displays percentage bias in the risk difference estimate when $\beta_{1}$ was set to log(1.5) and there were 30% missing outcome data. Unadjusted analysis was the most biased approach for the majority of covariate–outcome relationships, as expected given data were missing not at random under this approach. Adjusting for randomisation categories resulted in underestimation of the risk difference in all scenarios except for those involving a step function, while incorrectly assuming a linear relationship resulted in biased estimates for $f\left( X \right)=X^{2}$ but not $f\left( X \right)=e^{X}$ or $f\left( X \right)=X^{strat}$. As in the simulation study for continuous outcomes, both FP-2 and cubic splines were associated with minimal bias across all scenarios (maximum relative bias of 2.5%). As expected, the magnitude of bias was larger for strong covariate–outcome relationships. Estimates of the conditional log odds ratio also deviated from the true value of log(1.5) under misspecification of the covariate–outcome relationship with missing outcome data (results not shown), a combination of bias due to inadequate handling of missing data and estimation of a different conditional OR under model misspecification.

**Figure S4.** Percentage bias in scenarios with a binary outcome, 30% missing outcome data, treatment effect of log(1.5), and with standardisation to estimate the risk difference. The maximum Monte Carlo standard across all methods and scenarios was 0.99%.

**Example analysis of the DINO trial**

**Figure S5.** Relationship between birth weight and requirement for supplemental oxygen for chronic lung disease by 36 weeks’ gestation in the DINO trial. The estimated log odds for supplemental oxygen according to weight was obtained using lowess smoothing. Due to small numbers of infants at the extremes, to aid interpretation the plots have been truncated at the 5th and 95th percentiles of birth weight.
